# Do We Need to Add the Type of Treatment Planning System, Dose Calculation Grid Size, and CT Density Curve to Predictive Models?

**DOI:** 10.3390/diagnostics15060786

**Published:** 2025-03-20

**Authors:** Reza Reiazi, Surendra Prajapati, Leonardo Che Fru, Dongyeon Lee, Mohammad Salehpour

**Affiliations:** Department of Radiation Physics, Division of Radiation Oncology, The University of Texas MD Anderson Cancer Center, Houston, TX 77030, USA; sprajapati1@mdanderson.org (S.P.); lche@mdanderson.org (L.C.F.); dlee18@mdanderson.org (D.L.); msalehpour@mdanderson.org (M.S.)

**Keywords:** treatment planning, predictive modeling, domain dependency, CT density curve, calculation grid

## Abstract

**Background:** Generalizability and domain dependency are critical challenges in developing predictive models for healthcare, particularly in medical diagnostics and radiation oncology. Predictive models designed to assess tumor recurrence rely on comprehensive and high-quality datasets, encompassing treatment planning parameters, imaging protocols, and patient-specific data. However, domain dependency, arising from variations in dose calculation algorithms, computed tomography (CT) density conversion curves, imaging modalities, and institutional protocols, can significantly undermine model reliability and clinical utility. **Methods:** This study evaluated dose calculation differences in the head and neck cancer treatment plans of 19 patients using two treatment planning systems, Pinnacle 9.10 and RayStation 11, with similar dose calculation algorithms. Variations in the dose grid size and CT density conversion curves were assessed for their impact on domain dependency. **Results:** Results showed that dose grid size differences had a more significant influence within RayStation than Pinnacle, while CT curve variations introduced potential domain discrepancies. The findings underscore the critical role of precise and standardized treatment planning in enhancing the reliability of predictive modeling for tumor recurrence assessment. **Conclusions:** Incorporating treatment planning parameters, such as dose distribution and target volumes, as explicit features in model training can mitigate the impact of domain dependency and enhance prediction accuracy. Solutions such as multi-institutional data harmonization and domain adaptation techniques are essential to improve model generalizability and robustness. These strategies support the better integration of predictive modeling into clinical workflows, ultimately optimizing patient outcomes and personalized treatment strategies.

## 1. Background

Quantitative image analysis has emerged as a valuable tool in radiation oncology, enabling the extraction of imaging features to predict treatment outcomes, assess tumor characteristics, and guide clinical decision-making. The integration of dosimetric data with imaging-based predictive models has further advanced this field, offering insights into tumor biology and patient responses to therapy [1,2]. Additionally, image-based analysis has shown promise in the diagnosis of tumor recurrence, particularly after radiotherapy, by identifying subtle imaging features that differentiate true progression from pseudo-progression or post-treatment changes. Predictive modeling approaches have been explored for recurrence assessment in various cancers, including glioblastoma and head and neck cancers, by integrating imaging biomarkers with clinical and treatment data. For example, the texture analysis of magnetic resonance imaging and positron emission tomography/computed tomography (PET/CT) images has been used to distinguish residual or recurrent disease from treatment-induced necrosis, demonstrating encouraging accuracy in several studies [3].

Generalizability refers to the ability of a predictive model to maintain reliability and accuracy when applied to data from a new, independent cohort of patients [4,5]. If a model is not generalizable across different datasets, it may capture biases introduced by data generation and processing protocols rather than learning meaningful relationships between features and clinical outcomes. Studies have shown that when generalizability is not addressed, predictive models may exhibit performance degradation over time as healthcare practices and patient characteristics evolve [6].

The most effective way to improve model generalizability is to use large-scale multi-institutional datasets. However, while multi-institutional datasets are highly desirable for enhancing model performance, it is essential to take precautions to prevent domain dependency when using them. Healthcare settings can vary in terms of unobserved confounders, deployment environments, protocols, and data drift over time [7], resulting in a domain dependency that affects the output of a predictive model [8,9]. In radiation oncology, domain dependency means that variations in imaging parameters impact the robustness and performance of the predictive models built upon them. Domain dependency can have the same impact on prediction performance as low generalizability. Although domain dependency in a medical dataset, especially imaging data, can usually be prevented, some parameters, such as the collecting instruments, are part of the training data characteristics, and it is not possible to remove them. Thus, those parameters must be incorporated into the training process as features.

Radiation oncology is well positioned to benefit from advancements in predictive modeling, as these models can provide clinical insights by integrating complex treatment data, particularly radiation dose representation [10]. One area where predictive modeling has shown promise is in assessing treatment outcomes and evaluating the risk of radiation-induced complications. When developing models designed to predict treatment response across various medical fields, including radiation oncology, the quality and quantity of training data play a crucial role in ensuring model accuracy and reliability.

The accurate calculation of radiation dose in radiation oncology treatments plays a significant role in determining treatment response, as variations in dose distribution can lead to different clinical outcomes. The precise dose calculation and delivery are crucial factors in achieving optimal tumor control while minimizing damage to surrounding healthy tissues [11]. Other studies have highlighted the direct link between dose calculation accuracy and treatment response, underlining the importance of employing sophisticated algorithms and techniques to ensure precise dose delivery in radiation oncology treatments and optimize patient outcomes [11,12].

In this study, we evaluated the impact of the type of treatment planning system used on the dose calculation to determine if this parameter is important to consider when using a radiotherapy patient cohort for developing a predictive model. By conducting this comparative analysis, we aimed to comprehensively assess the impact of treatment planning system selection on dose calculations in head and neck radiation therapy planning. To minimize the effect of different dose calculation algorithms, we used two treatment planning systems with similar dose calculation algorithms. Our findings provide valuable insights into the potential differences and implications for clinical decision-making models when employing different treatment planning systems in radiotherapy practice.

## 2. Methods

In this study, we comprehensively compared the dose calculation algorithms (DCAs) of two widely used treatment planning systems, Pinnacle 9.10 (Philips Radiation Oncology Systems, Fitchburg, WI, USA) and RayStation 11 (RaySearch Medical Laboratories AB, Stockholm, Sweden). Both treatment planning systems are specifically designed for radiation therapy treatment planning and employ a collapsed cone convolution—type DCA.

A patient cohort consisting of 19 individuals with standard head and neck cancer treatment plans was included in this study following approval from the Institutional Review Board. The patients were chosen to represent a diverse range of tumor characteristics and anatomical variations commonly encountered in clinical practice.

To investigate the impact on the dose calculations of different dose calculation grid sizes, two grid size options were employed: 1 mm and 3 mm. The smaller grid size allows for higher spatial resolution, potentially capturing finer details, while the larger grid size reduces computation time without sacrificing overall accuracy.

Additionally, two different CT density curves were utilized to assess their influence on dose calculations. CT density curves reflect the relationship between Hounsfield unit (HU) values from the CT scan and tissue density. By applying different curves, variations in tissue density mapping can be evaluated, which may affect dose calculation accuracy.

For each patient, the previously generated Pinnacle treatment plan was transferred to RayStation for dose recalculation. This approach ensured a direct comparison between the two treatment planning systems, with consistent patient anatomy and treatment parameters.

To minimize discrepancies in the delineation of regions of interest (ROIs) between the two systems, special care was taken in the definition of treatment volumes. The external contour in RayStation was extended to encompass the patient support structures, aligning with the definition used in Pinnacle. This ensured consistency in the ROI delineation process and minimized potential differences resulting from variations in contouring.

Various dose–volume histogram (DVH) parameters were extracted and analyzed to assess the dose calculation differences between the two treatment planning systems. DVH provides information about the distribution of radiation dose within specific structures. In this study, we focused on the following parameters:

- D99%: The dose received by 99% of the volume of a specific organ or tumor. It represents an extremely low dose threshold within the structure.
- D98%: The dose received by 98% of the volume of a specific organ or tumor. It represents a very low dose threshold within the structure.
- D95%: The dose received by 95% of the volume of a specific organ or tumor. It represents a low dose threshold within the structure.
- D50%: The dose received by 50% of the volume of a specific organ or tumor. It represents an average dose threshold within the structure.
- D2%: The dose received by 2% of the volume of a specific organ or tumor. It represents a very high dose threshold within the structure.
- D1%: The dose received by 1% of the volume of a specific organ or tumor. It represents an extremely high dose threshold within the structure.

These parameters provide critical insights into the delivered dose distribution within organs-at-risk, such as the left and right parotids, larynx, spinal cord, and brainstem, and the gross tumor volume (GTV). By analyzing these parameters, we can evaluate the potential differences in dose calculations between the two treatment planning systems.

## 3. Results and Discussion

**Figure 1:**
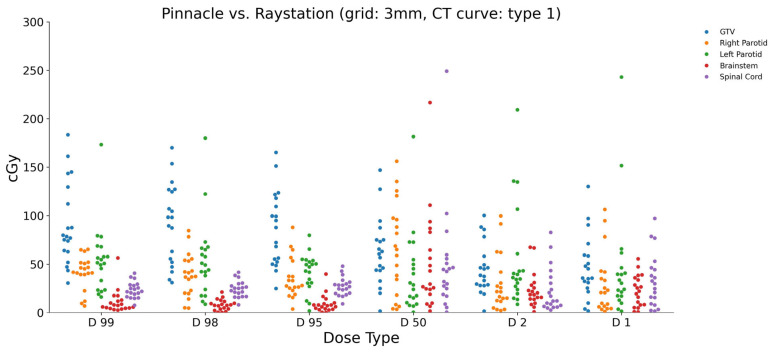
Calculated dose differences (cGy) between Pinnacle and RayStation using 3 mm grid size and CT curve type 1 for studied ROIs.

The calculated dose differences (in cGy) between Pinnacle and RayStation, using a 3 mm dose grid and CT curve type 1, for the studied ROIs are visualized in Figure 1. The minimum and maximum dose values are reported for each ROI. In the brainstem, the dose differences ranged from a minimum of 0.638 cGy to a maximum of 67.347 cGy for the different dose parameters (D1, D2, D50, D95, D98, and D99). Similarly, for the GTV, the dose differences varied from 1.442 cGy to 170.040 cGy across different dose parameters. The left and right parotids showed dose differences ranging from 0.959 cGy to 173.283 cGy and from 6.874 cGy to 65.198 cGy, respectively. The spinal cord exhibited dose differences between 1.477 cGy and 40.624 cGy.

**Figure 2:**
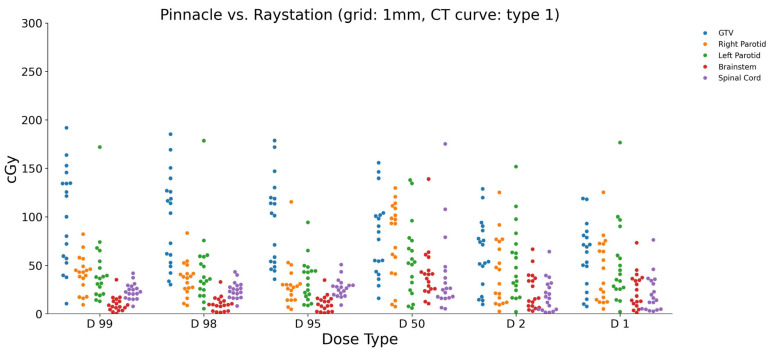
Calculated dose differences (cGy) between Pinnacle and RayStation using 1 mm grid size and CT curve type 1 for studied ROIs.

When the dose grid size was changed to 1 mm, the calculated dose differences between Pinnacle and RayStation again exhibited noticeable variations. Figure 2 provides a visual representation of the minimum and maximum dose values for each ROI using the 1 mm grid size and CT curve type 1. In the brainstem, the dose differences ranged from a minimum of 1.247 cGy to a maximum of 73.276 cGy for the different dose parameters. Similarly, for the GTV, the dose differences varied from 7.700 cGy to 191.767 cGy across the different dose parameters. The left and right parotids exhibited dose differences ranging from 2.160 cGy to 172.008 cGy and 5.089 cGy to 82.167 cGy, respectively. The spinal cord showed dose differences between 2.695 cGy and 41.875 cGy. These results clearly demonstrate the impact of changing the dose grid size on the calculated dose differences between the two treatment planning systems. The differences observed for each ROI and dose parameter indicate that the selection of the dose grid size can significantly influence the accuracy of the dose calculation.

**Figure 3:**
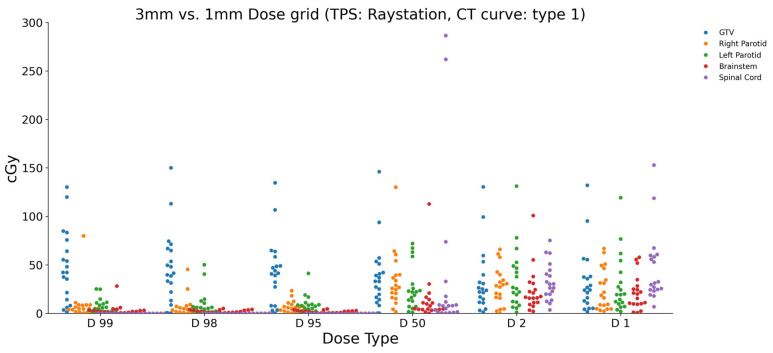
The calculated dose differences (cGy) in RayStation for two different dose grid sizes (1 mm vs. 3 mm) and a CT density curve type 1. The maximum and minimum dose differences observed were for the GTV (D50: 286 cGy) and spinal cord (D98: 0.05 cGy).

To investigate the impact of changing the dose grid size on a single treatment planning system, specifically RayStation, the grid size was adjusted from 1 mm to 3 mm. The resulting dose differences between the 3 mm and 1 bmm grid sizes demonstrate the minimum and maximum dose values for each ROI (Figure 3). For the brainstem, the dose differences ranged from a minimum of 0.733 cGy to a maximum of 100.841 cGy for the different dose parameters (D1, D2, D50, D95, D98, and D99). Similarly, for the GTV, the dose differences varied from 1.338 cGy to 150.044 cGy across the dose parameters. The left and right parotids exhibited dose differences ranging from 1.156 cGy to 25.139 cGy and 0.147 cGy to 79.911 cGy, respectively. The spinal cord showed dose differences between 3.512 cGy and 286.363 cGy. These results highlight the influence of changing the dose grid size solely within the RayStation treatment planning system. The observed variations for each ROI and dose parameter underscore the sensitivity of dose calculations to the grid size selection.

**Figure 4:**
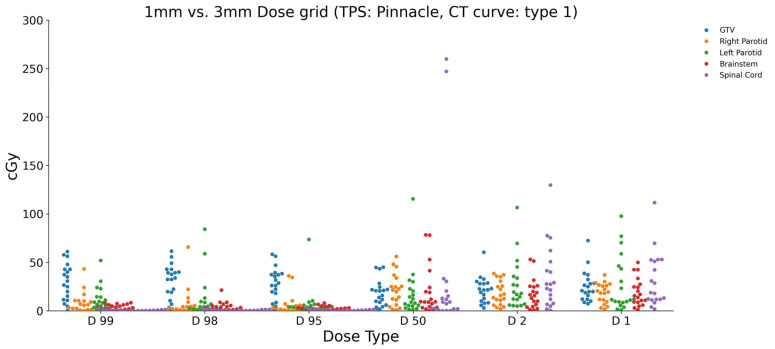
The calculated dose differences (cGy) in Pinnacle for 1 mm vs. 3 mm grid sizes and a CT density curve of type 1.

Similarly, the dose grid size was modified from 1 mm to 3 mm within the Pinnacle treatment planning system. The resulting dose differences between the 3 mm and 1 mm grid sizes illustrate the minimum and maximum dose values for each ROI. Figure 4 provides a visual representation of these changes. For the brainstem, the dose differences ranged from a minimum of 1.073 cGy to a maximum of 52.947 cGy for the different dose parameters (D1, D2, D50, D95, D98, and D99). In the case of the GTV, the dose differences varied from 2.893 cGy to 61.492 cGy across the dose parameters. The left and right parotids exhibited dose differences ranging from 0.915 cGy to 11.839 cGy and 1.538 cGy to 43.198 cGy, respectively. The spinal cord showed dose differences between 0.432 cGy and 260 cGy.

These results highlight the influence of changing the dose grid size exclusively within the Pinnacle treatment planning system. The observed variations for each ROI and dose parameter emphasize the sensitivity of dose calculations to the grid size selection.

A comparison of the dose differences between the RayStation and Pinnacle treatment planning systems indicated that the variance in dose grid size had a larger impact on RayStation than on Pinnacle doses.

**Figure 5:**
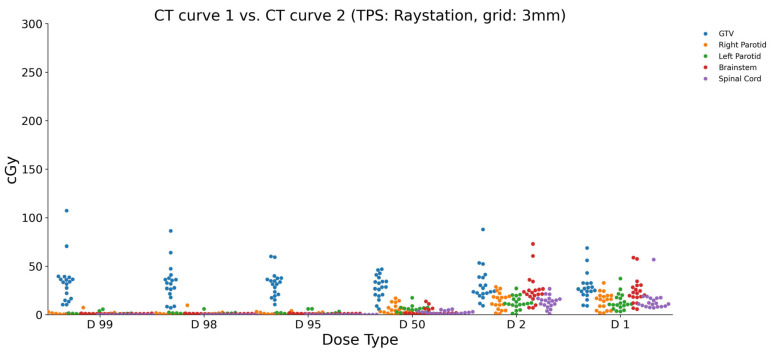
The calculated dose differences (cGy) in RayStation between the CT density curves type 1 and type 2 using a 3 mm grid size. The maximum and minimum differences observed were for the GTV (D99: 110 cGy) and the brainstem (D99: 0 cGy).

Lastly, to evaluate the impact of the CT curve on dose calculations within RayStation, the dose differences between type 1 and type 2 CT curves were analyzed (Figure 5). For the brainstem, the dose differences ranged from a minimum of 5.692 cGy to a maximum of 58.823 cGy across various dose parameters (D1, D2, D50, D95, D98, and D99). Similarly, the GTV showed dose differences ranging from 9.101 cGy to 107.235 cGy. The left and right parotids exhibited dose differences ranging from 1.080 cGy to 3.301 cGy and 1.668 cGy to 7.280 cGy, respectively. The spinal cord displayed dose differences between 0.012 cGy and 7.041 cGy.

Quantitative image analysis plays an increasingly important role in addressing challenges associated with distinguishing true progression from pseudo-progression following radiotherapy [13,14,15]. Advanced imaging techniques, including texture analysis and feature extraction, have been utilized to uncover patterns in imaging data that may not be visually apparent. These techniques are particularly valuable in integrating multi-modal data, such as genomics, histopathology, and imaging, along with treatment-related parameters like radiotherapy treatment planning [16,17,18,19], and hold great promise for improving the accuracy and robustness of recurrence prediction models [20,21]. Studies have demonstrated that combining imaging-based machine learning with genomic markers and radiotherapy dose distribution improves the performance of a clinical decision-making model in distinguishing true progression from pseudo-progression. While these applications have shown promise, variability in imaging protocols, feature extraction methods, and data acquisition remain significant barriers to the clinical implementation and reliability of these models [22,23,24,25]. Efforts to standardize feature extraction workflows, including initiatives like the Imaging Biomarker Standardization Initiative, are helping to reduce variability and improve reproducibility [26].

In this study, we analyzed the impact of two key factors in dose calculation—dose grid size and CT density curve—to investigate potential domain dependency in dosimetric data. Specifically, we examined how variations in treatment planning software or configuration influence dose calculations in radiotherapy treatment plans. Understanding these variations is essential for ensuring the reliability of predictive models that utilize dosimetric data for treatment outcome assessment. In a head and neck patient cohort consisting of 19 individuals, we compared the dose differences between two different treatment planning systems to simulate the multi-institutional environment. Specifically, we adopted the collapsed cone convolution method to calculate dose distributions to minimize external effects in the analysis of domain dependency. We used two dose grid sizes in the dose calculations and compared the dose differences using various DVH parameters. The ranges of dose differences for the ROIs were similar between 1 mm and 3 mm grid sizes and indicated that the dose grid size can affect the dose calculation. However, the size of the dose grid had only a small influence on the domain dependency when the dose calculation was performed through different treatment planning systems. The comparison of dose differences in a single treatment planning system indicated that the variance of the dose grid size had a larger impact on RayStation than on Pinnacle. Furthermore, the analysis of dose differences with different CT density conversion curves indicated that domain discrepancy may occur in RayStation when variant CT curves are applied.

## 4. Conclusions

In conclusion, the observed dose differences between treatment planning systems, as demonstrated in our study, have the potential to impact treatment response and the subsequent development of decision-making models. While the maximum dose difference may occur only in rare cases and be patient-dependent, it should not be disregarded as it can have practical implications.

To mitigate the impact of these discrepancies, it is essential to adhere to reporting guidelines such as the Transparent Reporting of a Multivariable Prediction Model for Individual Prognosis or Diagnosis (TRIPOD) [27]. Explicitly reporting the data collection tools and processes according to TRIPOD guidelines can establish a strong link between machine learning and clinical communities, facilitating a better understanding and interpretation of model performance. Explicitly reporting the data collection tools and processes according to TRIPOD guidelines can improve transparency and facilitate better understanding and interpretation of model performance. Moreover, adopting best practices from classical outcome prediction modeling—including prospectively registered study protocols, data analysis plans [28,29], and the publication of full models and code for independent validation—is crucial for ensuring transparency, reproducibility, and reliability in predictive modeling. These measures, previously highlighted in the QUANTEC papers, remain highly relevant in addressing challenges related to treatment planning and outcome prediction [10,30].

Additionally, considering the parameters involved in treatment planning—such as dose calculation algorithms, optimization techniques, planners, and contouring methods—is crucial. The success of the optimization process depends on the cost function used by the algorithm, the structures defined by the user, and the algorithm employed for minimization. The situation can be further exacerbated if different treatment planning parameters are used for the same patient, resulting in notable differences in treatment output even with the same prescribed dose. Therefore, ensuring consistency and standardization in these planning parameters is vital to achieving more reliable and comparable treatment outcome predictions.

By acknowledging the potential impact of dose differences, adhering to reporting guidelines, and considering the influence of treatment planning parameters, we can enhance treatment response assessments and improve the reliability of predictive models in radiation oncology. These steps will contribute to advancing the field and improving the integration of predictive modeling into clinical practice.

Future AI-driven studies, such as domain adaptation and multi-institutional data harmonization, may help address the impact of treatment planning variations on model performance. Investigating these techniques could provide further insights into improving generalizability and clinical applicability in outcome prediction models.

## Data Availability

The original contributions presented in this study are included in the article. Further inquiries can be directed to the corresponding author.
